# Lateral and Longitudinal Driving Behavior Prediction Based on Improved Deep Belief Network

**DOI:** 10.3390/s21248498

**Published:** 2021-12-20

**Authors:** Lei Yang, Chunqing Zhao, Chao Lu, Lianzhen Wei, Jianwei Gong

**Affiliations:** 1 School of Mechanical Engineering, Beijing Institute of Technology, Beijing 100081, China; 3120170237@bit.edu.cn (L.Y.); chaolu@bit.edu.cn (C.L.); 3120200396@bit.edu.cn (L.W.); 2 China North Vehicle Research Institute, Beijing 100072, China; zhaocq2013@163.com; 3 Yangtze Delta Region Academy of Beijing Institute of Technology, Jiaxing 314019, China

**Keywords:** driving behavior prediction, deep belief network, intelligent vehicles

## Abstract

Accurately predicting driving behavior can help to avoid potential improper maneuvers of human drivers, thus guaranteeing safe driving for intelligent vehicles. In this paper, we propose a novel deep belief network (DBN), called MSR-DBN, by integrating a multi-target sigmoid regression (MSR) layer with DBN to predict the front wheel angle and speed of the ego vehicle. Precisely, the MSR-DBN consists of two sub-networks: one is for the front wheel angle, and the other one is for speed. This MSR-DBN model allows ones to optimize lateral and longitudinal behavior predictions through a systematic testing method. In addition, we consider the historical states of the ego vehicle and surrounding vehicles and the driver’s operations as inputs to predict driving behaviors in a real-world environment. Comparison of the prediction results of MSR-DBN with a general DBN model, back propagation (BP) neural network, support vector regression (SVR), and radical basis function (RBF) neural network, demonstrates that the proposed MSR-DBN outperforms the others in terms of accuracy and robustness.

## 1. Introduction

Intelligent transportation systems aim to improve traffic efficiency, safety, and driver comfort in various situations [1,2,3]. As an essential element of intelligent transportation systems, intelligent vehicles have potentials to help traffic participants make effective decisions for driving safely and efficiently. Most driver assistance systems are developed to ensure and improve driving safety in specific critical environments [1], which requires a clear and comprehensive understanding of driving behavior to achieve a high level of intelligence [4]. An accurate driving behavior prediction can leave sufficient time for driver assistance systems to deliver a warning to the driver or for autonomous systems to directly take over the vehicle to ensure traffic safety [1]. Many researchers focus on driving behavior prediction regarding elements, such as motion, gaze behavior, and intention [5,6,7,8]. Ref. [9] proposed a new evolving fuzzy systems method based on adaptive fuzzy pattern classification for the detecting the lane-change intention of a driver. Ref. [10] applied non-linear polynomial regression and recurrent hidden semi-Markov model to realize the recognition of driver lane-change intention. Here, we focus on predicting driving behaviors such as lateral and longitudinal behaviors. Some mature model-based methods have been used for prediction such as dynamic Bayesian networks, support vector machine, and hidden Markov models [11,12,13,14]. For example, Kumagai presents a driving behavior prediction method based on dynamic Bayesian networks and shows good prediction results for stop behavior [15]. A prediction approach based on a dynamic Bayesian network is proposed for lane-change maneuvers and also shows good accuracy [16]. Ref. [17] develops a prediction method for lane-change maneuver of the vehicle ahead by using a hidden Markov model, and result shows that the model can achieve a high accuracy rate. However, model-based methods cannot adapt to the infinite complexity of driving behaviors.

Fortunately, the data-driven methods, such as artificial neural networks (ANNs), have been widely used for driving behavior prediction because of their flexible structures and powerful capability to describe non-linearity [18,19,20,21]. For instance, ref. [19] used three variables (safe speed, workload, and yaw rate) as the inputs to predict a safe speed in curve negotiation based on a two-layer back propagation (BP) neural network. In addition, the ANNs were also used to predict upcoming lane change behavior by considering three phases including lane change intention, preparation, and action [20]. Although significant achievements have been made, the existing ANN-based methods, such as BP and Radial Basis Function (RBF) neural network showed a limited accuracy in driving behavior learning and prediction because of their shallow architecture and manually-selected features [19,22,23].

The advanced on-board sensors allow obtaining plentiful data, which enables some data-hungry techniques to be practicable such as deep learning. Deep learning has attracted full attention because of its capability to automatically and genuinely extract features [24,25,26,27]. Many recent works on driving behavior prediction are primarily based on the image or video data from cameras [28,29,30,31]. For instance, researchers of [1,32] used raw images and human driving videos from camera to predict different driver actions including steering and braking/acceleration based on a recurrent neural network (RNN). Ref. [33] exploited a convolutional neural network (CNN) to extract features in a scene understanding subsystem for decision-making with considering the features from images. Ref. [34] proposed a novel method to predict lane-change maneuvers in highway scenarios by using deep learning and visual representations of the traffic scene. Additionally, some scholars studied driving behavior using mobile-based signals, for example, ref. [35,36,37]. However, the majority research above relied on image data rather than focused on the deep end-to-end network based on the data from on-board sensors for driving behavior prediction. CNN is also widely used in a wide range of image-related research work due to its powerful convolution structure in image feature extraction and recognition. It is suitable for dealing with 2-D data such as image data, but not suitable for working on the data obtained from on-board sensors [38].

Some existing works used the real-time and historical data collected from on-board sensors to predict driving behaviors [39]. For example, ref. [26] took road geometry, historical traffic information, and driver specific speed as inputs of a deep network stacked autoencoder to predict current vehicle speed. Based on the historical sensory data collected from natural driving, the structural RNN can predict the forthcoming lane-change behaviors of surrounding vehicles in 1∼3 s ahead [27]. Ref. [40] proposed a new model to predict the lane-change maneuvers by considering the historical information and neighbor vehicles’ information. Driving behavior prediction should be not only based on the understanding of traffic information but also based on the learning of driving control behavior of human drivers to realize a high level of intelligence. However, the research above did not take both human driver actions and environmental behaviors into consideration to predict the future behavior of the ego vehicle.

Although the deep learning models can extract more features than shallow ones, the above existing methods only separately predict longitudinal and lateral behaviors. For example, ref. [26] focuses on the longitudinal while [27] focuses on lane change behavior. Ref. [1] predicted lane change, steering, acceleration, and braking, but the predictions of longitudinal and lateral behaviors are conducted regardless of their mutual relationship. Among all deep learning methods, the deep belief network (DBN) is relatively effective [41], and most of the applications based on DBN were classification [42]. Then, researchers applied DBN into traffic flow prediction [43]. For example, ref. [41] predicted the traffic flow using DBN with multitask learning, which proves the deep model can achieve much high prediction accuracy for traffic flow. Considering the ability of DBN to encode network with multiple layers and avoid over-fitting and local minimum [26,41,44], this paper attempts to develop a deep structure to predict longitudinal and lateral driving behaviors simultaneously.

In addition, the driving action implemented by the driver does depend on the states of surrounding vehicles. However, the future states of surrounding vehicles are unavailable when predicting the driver’s forthcoming behavior of the ego vehicle. Therefore, most behavior prediction methods are based on historical data without considering the future states of surrounding vehicles [1,32,33]. In this paper, we predict future states of surrounding vehicles by utilizing corresponding historical states. Then, we predict the future maneuver of the ego vehicle based on predicted states of surrounding vehicles and historical multi-sensor data including the states of surrounding vehicles, the ego vehicle, and driver control.

Tackling the problem of behavior prediction can simplify the problem of trajectory prediction and contribute to the decision-making of the vehicle [27]. From this perspective, this paper demonstrates that the deep learning model based on DBN can extract features to predict and understand the driving behavior and then utilizes the DBN to predict the lateral and longitudinal behaviors of an ego vehicle.

**Figure 1:**
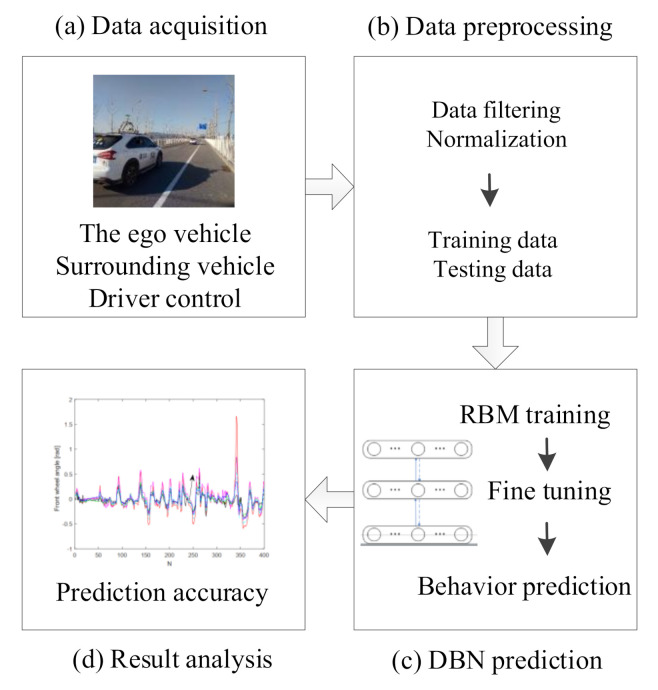
Proposed driving behavior prediction system.

Figure 1 illustrates our proposed driving behavior prediction system, which consists of four modules: data acquisition, data preprocessing, DBN prediction, and result analysis. The main contributions of this paper are in three-fold:

- Developing a general prediction system, which allows us to consider real-world data including states of surrounding vehicles and the ego vehicle and the driver’s control inputs simultaneously to predict the driving behavior in an end-to-end way;
- Proposing a systematic testing method to obtain optimal parameters of the prediction model;
- Proposing an MSR-DBN prediction model with a multi-target sigmoid regression layer to realize coupled optimization for lateral and longitudinal behavior prediction.

The remainder of the paper is organized as follows. Section 2 presents the DBN prediction architecture and proposes the MSR-DBN prediction model. Section 3 presents the data collection and data pre-training for the experiment. Section 4 shows the experiment process of determining the optimal structure of our proposed model. Then, the prediction results based on MSR-DBN and comparison results are analyzed. Section 5 gives further discussion and conclusion.

## 2. Driving Behavior Prediction Model

### 2.1. MSR-DBN Prediction Model

**Figure 2:**
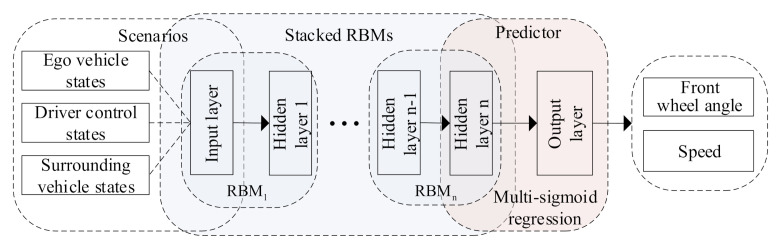
The typical DBN driving behavior prediction architecture.

The DBN, as a deep learning approach, usually is composed of several restricted Boltzmann machines (RBMs) [45,46]. Figure 2 displays the typical DBN prediction architecture with a multi-target sigmoid regression (MSR) layer as the predictor. The architecture is composed of one input layer, *n* hidden layers (i.e., *n* RBMs), and one output layer. The input layer is the first RBM (denoted as ${\mathrm{RBM}}_{1}$), which is observable.

Our goal is to predict the lateral and longitudinal behaviors of an ego vehicle such as the front wheel angle and speed by considering the behaviors of surrounding vehicles. To this end, we select three states as the model inputs, including the states of the ego vehicle, the driver operations, and the states of surrounding vehicles. Specifically, we denote the inputs as *I* = {$I_{E},I_{D},I_{S}$}, i.e., the states of the ego vehicle ($I_{E}$), driver control ($I_{D}$), and surrounding vehicles ($I_{S}$), respectively. These three inputs are defined as

(1) $$\begin{array}{c} \begin{array}{cc} I_{E} & =v_{(t-N):t}\\ I_{D} & =\alpha_{(t-N):t}\\ I_{S} & =[▵x_{\left(t-N):(t+1\right)},▵y_{\left(t-N):(t+1\right)},▵v_{\left(t-N):(t+1\right)}] \end{array} \end{array}$$

where t−N denotes the historical time t−N, *t* denotes the current moment, t+1 denotes the future moment; $v_{(t-N):t}$ and $\alpha_{(t-N):t}$ are a series of data for speed and front wheel angle of the ego vehicle from time t−N to *t*; $▵x_{\left(t-N):(t+1\right)}$, $▵y_{\left(t-N):(t+1\right)}$, and $▵v_{\left(t-N):(t+1\right)}$ are the relative lateral position, relative longitudinal position, and relative speed between the ego vehicle and surrounding vehicles from time t−N to t+1, respectively.

**Figure 3:**
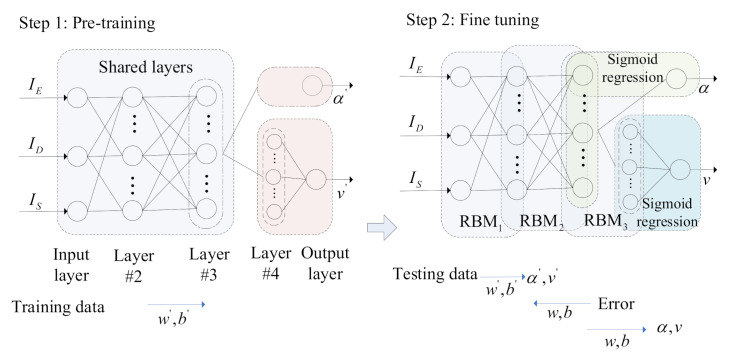
Improved MSR-DBN prediction model.

We conducted a series of experiments and found that traditional architecture in Figure 2 cannot obtain an expected prediction performance for both front wheel angle and speed simultaneously. One of the reason is that the model has an integrated structure, and the same fixed layers and model parameters are obtained and used to predict lateral and longitudinal behaviors simultaneously. However, these two behaviors are significantly different from each other. To overcome this limitation, we modify the DBN structure by adopting different sub-structures for front wheel angle (α) and speed (*v*) separately with the same input and output (Figure 3). Our proposed model contains two sub-networks: a four-layer sub-network for front wheel angle and a five-layer sub-network for speed, to extract features and predict the front wheel angle and speed of the ego vehicle (i.e., Step 1 in Figure 3). This modification makes it tractable to optimize the couplings of the lateral and longitudinal behaviors.

In order to improve the prediction efficiency, Layer #2 and Layer #3 share a sub-network between the front wheel angle and speed in the new model, i.e., ${\mathrm{RBM}}_{1}$ and ${\mathrm{RBM}}_{2}$ share the RBMs to train model parameters for two sub-networks (see Step 1 and Step 2 in Figure 3). The shared layers can not only simplify the model structure and reduce model parameters but also contribute to reducing the redundant computing burden. The output layer in the front wheel angle sub-network and the Layer #4 and output layer in speed sub-network are independent layers to extract further features and predict lateral and longitudinal behaviors. We chosen 100 hidden nodes in the shared layers (Layers #2 and #3) and Layer #4; see details in Section 4.

### 2.2. Training Procedure

Figure 3 shows the training process of our model including two steps [41,47,48,49]: pre-training and fine tuning. The algorithm of the proposed MSR-DBN prediction model is summarized as:

- Initialization—Initialize the weights and biases and preprocess training and testing data;
- Pre-training—Train the shared layers and independent layers for the feature extraction to obtain the model parameters $\left\{w^{\prime },b^{\prime }\right\}$ based on two sub-networks (i.e., Step 1 in Figure 3);
- Fine tuning—Obtain the initial prediction $\left\{\alpha^{\prime },v^{\prime }\right\}$ based on a multi-target sigmoid regression layer, and utilize a back propagation to fine tune the final prediction model (i.e., Step 2 in Figure 3);
- Prediction—Predict driving behavior and output {α,v} for the ego vehicle.

In addition, the pre-training procedure can produce the initial model parameters by the unsupervised learning, based on which the fine-tuning procedure operates the unsupervised learning to obtain the initially predicted behaviors. Then the supervised learning method is used to predict the behaviors. Note that, the pre-training trains the initial model parameters in an unsupervised method without given driving behavior labels, while the fine tuning adjusts and updates the parameters in a supervised method based on labeled behaviors. In what follows, we will detail the training and tuning procedures.

#### 2.2.1. Pre-Training Procedure

**Figure 4:**
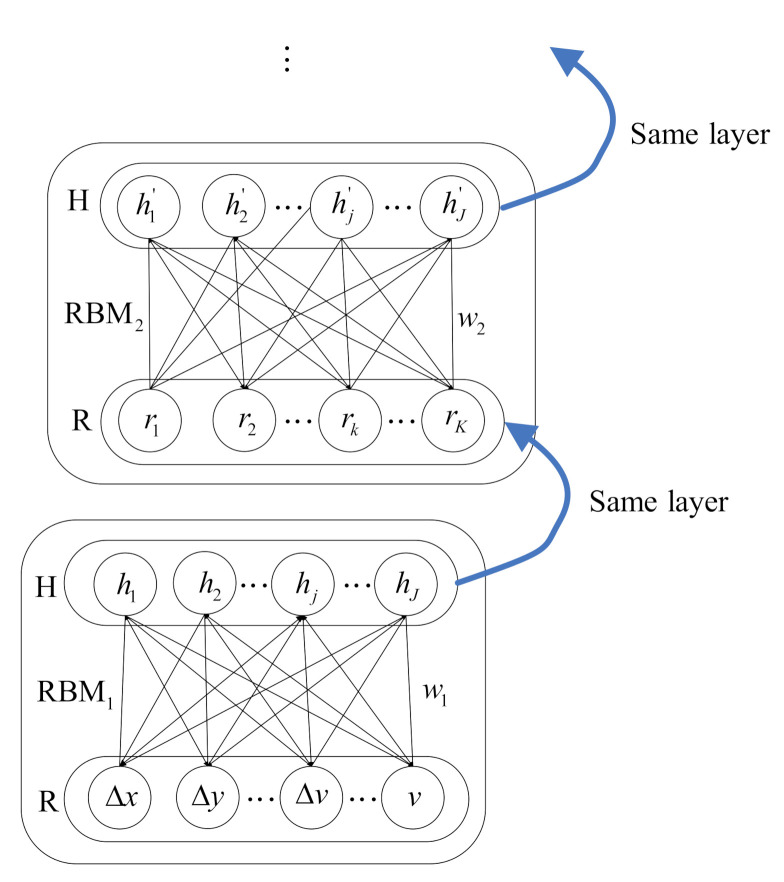
Schematic diagram for the RBM and training process for the pre-training.

Based on a stack of RBMs, pre-training is to initialize parameters through unsupervised greedy algorithms. Usually, the RBM is composed of a visible layer (denoted as R) and a hidden layer (denoted as H), as shown in Figure 4. ${\mathrm{RBM}}_{1}$ is the first RBM, and nodes ▵x, ▵y, ▵v, *v* represent the visible nodes of ${\mathrm{RBM}}_{1}$. The nodes between layers are interconnected while those in the same layer are disconnected with each other [41,50].

Define P(r,h|θ) as the probability distribution of a single RBM, then the energy function of the RBM is given as

(2) $$\begin{array}{c} \begin{array}{cc} E(r,h|\theta ) & =\ln P(r,h|\theta )\\ & =-\sum_{k=1}^{K}a_{k}r_{k}-\sum_{j=1}^{J}b_{j}h_{j}-\sum_{k=1}^{K}\sum_{j=1}^{J}w_{kj}r_{k}h_{j} \end{array} \end{array}$$

where $\theta =(w_{kj},a_{k},b_{j})$ are the model parameters, $w_{kj}$ is the weight from the *k*-th visible node $r_{k}$ to the *j*-th hidden node $h_{j}$; $a_{k}$ and $b_{j}$ are the biases of $r_{k}$ and $h_{j}$ in a single RBM, respectively. For the input layer in Figure 3, the input units including $I_{E}$, $I_{D}$, and $I_{S}$ correspond to the visible nodes $r_{k}$ of the R layer for ${\mathrm{RBM}}_{1}$, as shown in Figure 4 and Equation (2).

Given the state of the visible unit *r* and the state of the hidden unit *h*, the activation probabilities of $h_{j}$ and $r_{k}$ are written as

(3) $$\begin{array}{c} \begin{array}{cc} P(h_{j}|r,\theta ) & =\mathrm{sigm}(b_{j}+\sum_{k=1}^{K}r_{k}w_{kj})\\ P(r_{k}|h,\theta ) & =\mathrm{sigm}(a_{k}+\sum_{j=1}^{J}h_{j}w_{kj}) \end{array} \end{array}$$

with the sigmoid function

(4) sigm(z)=1/(1+exp(−z))

We update the parameter θ using the contrastive divergence algorithm [45,51]. The update rules for weight $w_{kj}$ and biases $a_{k}$ and $b_{j}$ are given as

(5) $$\begin{array}{cc} ▵w_{kj} & =\eta \cdot (E{\left(r_{k}h_{j}\right)}_{\mathrm{data}}-E{\left(r_{k}h_{j}\right)}_{\mathrm{model}})\\ ▵a_{k} & =\eta \cdot (E{\left(r_{k}\right)}_{\mathrm{data}}-E{\left(r_{k}\right)}_{\mathrm{model}})\\ ▵b_{j} & =\eta \cdot (E{\left(h_{j}\right)}_{\mathrm{data}}-E{\left(h_{j}\right)}_{\mathrm{model}}) \end{array}$$

where η is the learning rate for pre-training procedure which varies from zero to one, $E{\left(\cdot \right)}_{\mathrm{data}}$ and $E{\left(\cdot \right)}_{\mathrm{model}}$ represent the expectations of the training data and the distribution of the model, respectively.

In the DBN, the output of an RBM is the input of the next RBM, that is, the adjacent RBMs have a shared layer with the same nodes, as shown in Figure 4. The pre-training process is achieved via Step 1 in Figure 3. All the RBMs are trained one by one. Specifically, the RBMs are trained from the first RBM to the last one, and once an RBM is trained, its output will be regarded as the input of the next RBM. Thus, we can initialize the model parameters $\left\{w^{\prime },b^{\prime }\right\}$ for the fine-tuning procedure. Then, the output of the last RBM is fed as the input of the sigmoid regression (Figure 2 and Figure 3).

#### 2.2.2. Fine-Tuning Procedure

The fine-tuning procedure is a supervised part based on BP network, and Step 2 in Figure 3 explains the training process. Once obtaining the initial weights and biases for the prediction model after training RBMs one by one, the fine tuning adjusts model parameters to obtain the final prediction model.

Specifically, after obtaining the initial parameters $\theta^{\prime }=\left(w_{kj}^{\prime },a_{i}^{\prime },b_{j}^{\prime }\right)$ through the pre-training procedure, the network makes use of the forward propagation algorithm to compute the output of the hidden units.Then, a multi-target sigmoid regression layer is applied as shown in Figure 2 to predict the initial output $O_{p}^{\prime }$ (i.e., initial front wheel angle and speed) [41]. The initial output $O_{p}^{\prime }=\left\{O_{p_{\alpha }}^{\prime },O_{p_{v}}^{\prime }\right\}$ of the multi-target sigmoid regression layer is defined as

(6) $$\begin{array}{cc} O_{p_{\alpha }}^{\prime } & =\mathrm{sigm}(b_{\alpha }^{\prime }+{O_{p_{\alpha }}^{\prime }}_{-1}w_{\alpha }^{\prime })\\ O_{p_{v}}^{\prime } & =\mathrm{sigm}(b_{v}^{\prime }+{O_{p_{v}}^{\prime }}_{-1}w_{v}^{\prime }) \end{array}$$

where {$b_{\alpha }^{\prime }$,$w_{\alpha }^{\prime }$} and {$b_{v}^{\prime }$,$w_{v}^{\prime }$} are the initial biases and weights for $\alpha^{\prime }$ (i.e., $O_{p_{\alpha }}^{\prime }$) and $v^{\prime }$ (i.e., $O_{p_{v}}^{\prime }$), respectively. $O_{p}^{\prime }$ is the output for the output layer of Step 1 in Figure 3, and ${O_{p_{a}}^{\prime }}_{-1}$ and ${O_{p_{s}}^{\prime }}_{-1}$ are the initial outputs of Layer #3 and Layer #4 for front wheel angle and speed, as shown in Figure 3.

In order to optimize the prediction model, the BP network uses a back propagation algorithm to fine tune the parameters $\theta^{\prime }$. We need to calculate the sensitivity σ of layers to modify the parameter of the model from the top layer to the bottom layer. For the predictor layer, the predicted output for the *k*-th node is assumed to ${O_{p}^{\prime }}_{k}$, and the actual expected output is ${O_{a}^{\prime }}_{k}$. The sensitivity σ can be computed by

(7) $$\begin{array}{c} \sigma_{k}={O_{p}^{\prime }}_{k}\left(1-{O_{p}^{\prime }}_{k}\right)\left({O_{a}^{\prime }}_{k}-{O_{p}^{\prime }}_{k}\right) \end{array}$$

For the *l*-th hidden layer, σ can be written as

(8) $$\sigma_{k}^{l}={O_{p}^{\prime }}_{k}^{l}\left(1-{O_{p}^{\prime }}_{k}^{l}\right)\sum_{j}w_{kj}^{l}\sigma_{j}^{l+1}$$

Then, $\theta^{\prime }$ can be adjusted to θ and back to each layer. Thus, the model parameters can be updated and adjusted. Similar to Equation (6), the final output $O_{p}$, the prediction result of the model, is as below

(9) $$O_{p}=\left\{\alpha_{t+1},v_{t+1}\right\}$$

where $\alpha_{t+1}$ and $v_{t+1}$ are the final front wheel angle and speed of the ego vehicle at the future moment, respectively.

## 3. Data Collection and Data Processing

### 3.1. Data Collection

#### 3.1.1. Data Acquisition

We collected the real-vehicle data by BYD Surui, an autonomous vehicle of Intelligent Vehicle Research Center of Beijing Institute of Technology (BIT). The data acquisition platform was equipped with on-board sensors to collect vehicle and traffic data. An OxTs integrated navigation system was used to collect GPS information, such as latitude/longitude, heading angle, and time stamp of data. Vehicle CAN-bus provided throttle pedal position, brake pressure, front wheel angle, vehicle speed, and time stamp of data. Radar detected both mid-range and long-range obstacles and provided positions of states of surrounding vehicles. Additionally, a camera was used to collect the scene image information. Table 1 shows some important information of data collection.

**Figure 5:**
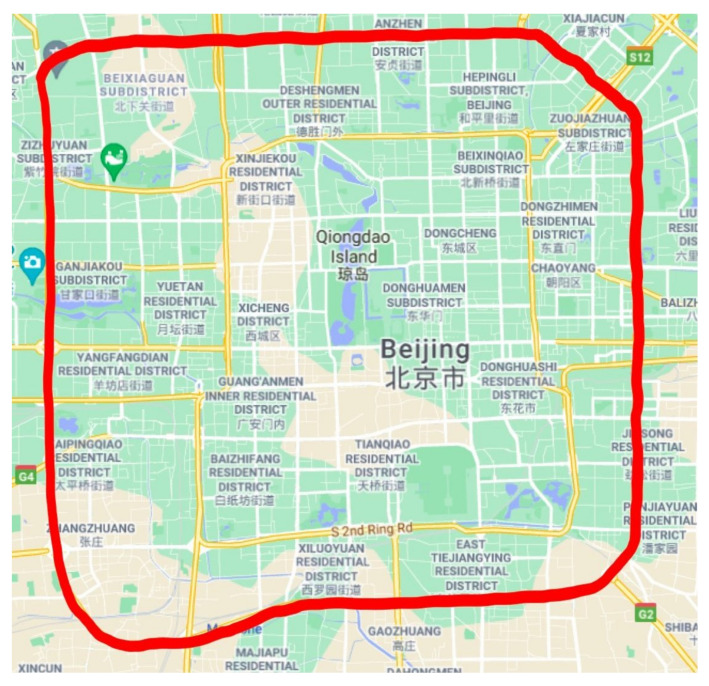
Data acquisition route.

Our vehicle was driven by an experienced human driver and interacted with surrounding vehicles in the Third Ring Road in Beijing, and the route is shown by the red curve in Figure 5. We focus on the naturalistic driving scene in the straight road rather than complex scenarios, such as turning scenario and intersection scenario. Specifically, we excluded the data under complex scenarios based on the scene images captured by the camera and selected the data. Finally, 20,000 samples were selected for the prediction.

#### 3.1.2. Data Preprocessing

We focus on the surrounding vehicles that have significant effects on the ego vehicle and extract the data of three surrounding vehicles including the left front vehicle, the front vehicle, and the right front vehicle. Based on the collected data of 64 target points by radar, we used dynamic target analysis and the geometric relationship, such as relative position and relative speed to calculate and extract the states of the surrounding vehicles. In addition, we extracted the historical speed (*v*) and front wheel angle (α) of the ego vehicle and the relative position (▵x and ▵y) and speed (▵v) of surrounding vehicles to train our prediction model. The historical front wheel angle of the ego vehicle are treated as the control state of the human driver. Thus, we integrated and preprocessed the driver’s operations, the states of the ego vehicle, and the states of surrounding vehicles. After obtaining the filtered data, we normalized them into [0, 1] to satisfy the input requirement of DBN, and then divided them into two groups as training and testing data.

### 3.2. Performance Evaluation

Three kinds of matrics are used to evaluate the performance of the prediction model by measuring the errors of prediction results [24,52,53,54], including root mean square error (RMSE),

(10) $$\mathrm{RMSE}={\left[\frac{1}{N}\sum_{n=1}^{N}{\left(O_{a}-O_{p}\right)}^{2}\right]}^{1/2}$$

mean absolute error (MAE),

(11) $$\mathrm{MAE}=\frac{1}{N}\sum_{n=1}^{N}\left|O_{a}-O_{p}\right|$$

and mean relative error (MRE),

(12) $$\mathrm{MRE}=\frac{1}{N}\sum_{n=1}^{N}\left|\frac{O_{a}-O_{p}}{O_{a}}\right|$$

where *N* represents the sample number, $O_{a}$ represents the observed actual behavior, $O_{p}$ represents the predicted behavior. Here, we do not use MRE to evaluate the prediction performance for the front wheel angle since the front wheel angle may be equal to 0 or close to 0, thus leading to a positive infinite value of MRE.

### 3.3. Model Input Selection

As mentioned above, this paper is based on a series of historical data and current states of the ego vehicle and surrounding vehicles to predict the driving behavior of the ego vehicle [55]. More than 20,000 samples are used to train and test our prediction model (the training data account for approximately 85%). The input includes the historical data of the ego vehicle, driver operation, and surrounding vehicles, as described in Equation (1). In urban complex traffic, the surrounding vehicle behaviors would affect the state of the ego vehicle since they need to interact with each other. Additionally, the future states of the surrounding vehicles will influence the decision-making of the ego vehicle. Therefore, we take the states of surrounding vehicles at the next moment as one of input to improve the prediction accuracy by simulating real scenarios. However, the future behaviors of surrounding vehicles are unknown when predicting the next state of the ego vehicle. Therefore, this section will firstly predict the driving states of surrounding vehicles.

We will explore different time-series inputs to find the appropriate one for the prediction model. A series of experiments demonstrate that the errors are relatively low when the learning rate η is 0.7, and the number of hidden nodes is 100, which will be introduced explicitly in Section 4. In this section, the learning rate and hidden nodes are similarly fixed (0.7 and 100, respectively) to show our experiment design clearly.

#### 3.3.1. State Prediction of Surrounding Vehicles

To simulate the real situation, we will predict the states of surrounding vehicles for the forthcoming moment. For the surrounding vehicles, the rear vehicles drive normally and have little influences on the ego vehicle which can be neglected, and we only consider the effects of front vehicles. Since increasing the number of layers of DBN would significantly increase the training time, we choose DBN with one hidden layer to predict the states of the surrounding vehicles.

Based on different historical data of surrounding vehicles, we can compute the errors of ▵x, ▵y, and ▵v (i.e., $▵x_{\mathrm{RMSE}}$, $▵x_{\mathrm{MAE}}$, $▵y_{\mathrm{RMSE}}$, $▵y_{\mathrm{MAE}}$, $▵v_{\mathrm{RMSE}}$, and $▵v_{\mathrm{MAE}}$) for surrounding vehicles including the left front vehicle, front vehicle, and right front vehicle, as shown in Table 2. Five cases (Case 00, Case 01, Case 02, Case 03, and Case 04) represent different historical data for previous 0 s, 1 s, 2 s, 3 s, and 4 s, respectively. In Table 2, the minimum errors for $▵x_{\mathrm{RMSE}}$, $▵x_{\mathrm{MAE}}$, $▵y_{\mathrm{RMSE}}$, $▵y_{\mathrm{MAE}}$, $▵v_{\mathrm{RMSE}}$, and $▵v_{\mathrm{MAE}}$ among three surrounding vehicles are in red; the second-minimum errors are in blue; the value in red in the type column is the selected type for our prediction model.

Table 2 indicates that Case 01 obtains the lowest $▵v_{\mathrm{RMSE}}$ and $▵v_{\mathrm{MAE}}$ for three surrounding vehicles among all types, and the second minimum for the majority $▵y_{\mathrm{RMSE}}$ and $▵y_{\mathrm{MAE}}$. Even though some $▵x_{\mathrm{RMSE}}$ and $▵x_{\mathrm{MAE}}$ in Case 01 do not obtain the lowest errors among all the five types, the relative lateral positions have little effects on the ego vehicle and all the vehicles driving regularly and safely on their own lanes, so we select Case 01 as the optimal one to predict surrounding vehicles in this paper. Thus, we choose Case 01 (i.e., the historical data 1 s ahead) to predict the states of surrounding vehicles at the next moment.

**Figure 6:**
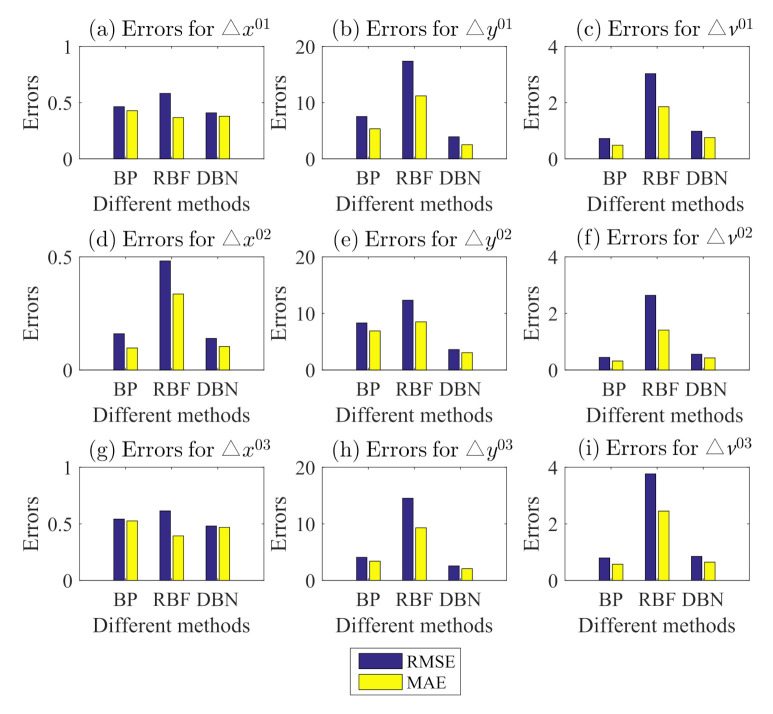
Prediction errors for surrounding vehicles based on different methods.

Figure 6 presents the comparison results with BP and RBF to illustrate the prediction accuracy for surrounding vehicles based on DBN, where superscripts 01, 02, and 03 represent the relative states of the left front vehicle ($vehicle_{1}$), front vehicle ($vehicle_{2}$) and right front vehicle ($vehicle_{3}$) with respect to the ego vehicle ($vehicle_{0}$), associated with Table 2. Most RMSE and MAE values of ▵x, ▵y and ▵v for three surrounding vehicles are slightly low, compared to those of BP and RBF. Thus, we utilize the prediction results of the surrounding vehicles to improve the driving state prediction for the ego vehicle.

#### 3.3.2. Different Historical Data

To obtain an optimal prediction performance of driving behavior for the ego vehicle, we test different inputs with different historical data of the ego vehicle and surrounding vehicles. Table 3 shows the prediction errors of α and *v* of DBN with one RBM (0.7 learning rate and 100 hidden layer nodes), where Case 0, Case 1, Case 2, Case 3, and Case 4 represent N=0,N=1,N=2,N=3, and N=4 in Equation (1), respectively. In order to describe the results efficiently, we term the prediction errors of the front wheel angle as $\alpha_{\mathrm{RMSE}}$ and $\alpha_{\mathrm{MAE}}$. Additionally, the RMSE, MAE, and MRE of the speed are abbreviated as $v_{\mathrm{RMSE}}$, $v_{\mathrm{MAE}}$, and $v_{\mathrm{MRE}}$.

Table 3 shows that Case 0 obtains the lowest $v_{\mathrm{RMSE}}$, $v_{\mathrm{MAE}}$ and $v_{\mathrm{MRE}}$ while Case 4 and 2 obtain the lowest $\alpha_{\mathrm{RMSE}}$, and $\alpha_{\mathrm{MAE}}$. The errors of speed are relatively higher than those of front wheel angle, so we consider the errors of speed in the first place. Even though Case 0 obtain the lowest value of the errors of speed, it obtains a more significant error of front wheel angle, compared to Case 4. Thus, we choose Case 1 since most of the errors of speed and front wheel angle are acceptable. The rest of this paper will regard the current states and historical states 1 s ahead of surrounding vehicles and the ego vehicle and the predicted states of surrounding vehicles at next moment as input to predict the driving behavior of the ego vehicle.

## 4. Results

### 4.1. Experiment of DBN Structure

Previous studies have obtained some prediction models based on DBN by determining different parameters, such as the prediction model for traffic flow [41]; however, it is still unknown which kind of structure is appropriate for driving behavior prediction, and there is not a general method to guide the model design. To obtain an optimal DBN structure for prediction, we explore a systematic testing method and train the model using the cut-and-try method which is similar to the training method used by [54]. Specifically, the performance of one parameter of the model is investigated by keeping other parameters fixed. The parameters of our prediction model mainly include the learning rate, the number of hidden layers, and the number of nodes in hidden layers [56,57]. The prediction model has 31 input nodes and 2 output nodes as described above.

#### 4.1.1. Learning Rates

The number of hidden layers and hidden nodes are determined by firstly testing different learning rates. In this subsection, we set the DBN structure with one hidden layer (i.e., one RBM) consisting of 100 hidden nodes. The prediction results of our model for different learning rates are obtained based on training data, as shown in Table 4. Both $\alpha_{\mathrm{MAE}}$ (0.0939) and $\alpha_{\mathrm{RMSE}}$ (0.0493) reach the best performance at the learning rate 0.5, and $v_{\mathrm{RMSE}}$, $v_{\mathrm{MAE}}$, and $v_{\mathrm{MRE}}$ obtain the best performance with the learning rate 0.9, respectively. However, a large learning rate would cause a poor prediction performance because of over-fitting [51]. Additionally, Table 4 demonstrates that the error of speed is larger than that of the front wheel angle. Thus, we chose the learning rate as 0.7 since all the errors are close to their associated lowest ones.

#### 4.1.2. Hidden Layers

The number of hidden layers is explored by fixing the number of hidden nodes in each hidden layer to 100. Table 5 shows the errors based on different hidden layers. The limited training data size causes an unsatisfied prediction performance of complex structure with over 3 layers for front wheel angle and speed, which may be caused by under-fitting. When the number of RBM is 3, all the prediction errors of speed ($v_{\mathrm{RMSE}}$ of 0.3858, $v_{\mathrm{MAE}}$ of 0.2965, and $v_{\mathrm{MRE}}$ of 0.0531) are the lowest values, i.e., it achieves the best performance. $\alpha_{\mathrm{RMSE}}$ and $\alpha_{\mathrm{MAE}}$ are the lowest values when setting the hidden layers as two and one, but larger errors for speed than those of three layers. For the three-hidden-layer structure, $\alpha_{\mathrm{RMSE}}=0.1431$ is slightly larger than the average error of 0.1290, but $\alpha_{\mathrm{MAE}}=0.0811$ is lower than the average value of 0.0862. In addition, this structure performs the best for speed as mentioned above. Therefore, we choice the hidden layer as 3.

#### 4.1.3. Hidden Nodes

Based on the analysis above, we test the model with different hidden nodes when the learning rate is 0.7, and the hidden layer is 3. The number of hidden nodes is set to 32, 50, 64, 100, 128, 150, 200, and 256, respectively. Table 6 shows the test results and indicates that for the front wheel angle, fewer hidden nodes in each hidden layer can achieve better performance. Specifically, $\alpha_{\mathrm{RMSE}}$ and $\alpha_{\mathrm{MAE}}$ obtain the lowest errors for the front wheel angle when the hidden nodes are [32, 32, 32] and [64, 64, 64]. For the speed, the structure with 100 hidden nodes in each layer is the best choice. Although $\alpha_{\mathrm{RMSE}}$ and $\alpha_{\mathrm{MAE}}$ are 0.1431 and 0.0811 with 100 hidden nodes, and are slightly larger than the lowest value, they are still lower than the average ones. However, inappropriate hidden nodes (e.g., 32 and 200) for the speed would lead to an unsatisfied performance because too few nodes are insufficient to extract representative features and too many nodes would lead to extra burden to the training model. Therefore, we set 100 nodes in each hidden layer.

Finally, we can obtain a three-hidden-layer DBN prediction model with 100 hidden nodes and the learning rate of 0.7, denoted as ${\mathrm{DBN}}_{3}^{100}$. The subscript represents the number of hidden layers and superscript represents the number of nodes in each hidden layer.

### 4.2. Structure of MSR-DBN

Based on the obtained ${\mathrm{DBN}}_{3}^{100}$, we should optimize the prediction performance further. Table 5 and Table 6 show that the ${\mathrm{DBN}}_{3}^{100}$ performs best for speed, but not for the front wheel angle. In addition, the two-hidden-layer structure with 100 nodes in Table 5 achieves much better performance for the front wheel angle, which is even better than the three-hidden-layer model with 32 and 64 nodes.

Considering both front wheel angle and speed, we modify the prediction model ${\mathrm{DBN}}_{3}^{100}$ and proposes a novel MSR-DBN prediction model which is a four-five-layer prediction model for the driving behavior of the ego vehicle as shown in Figure 3. Thus, the MSR-DBN consists of two sub-systems: a two-hidden-layers sub-network for front wheel angle and a three-hidden-layers sub-network for speed.

### 4.3. Result Analysis and Comparison

**Figure 7:**
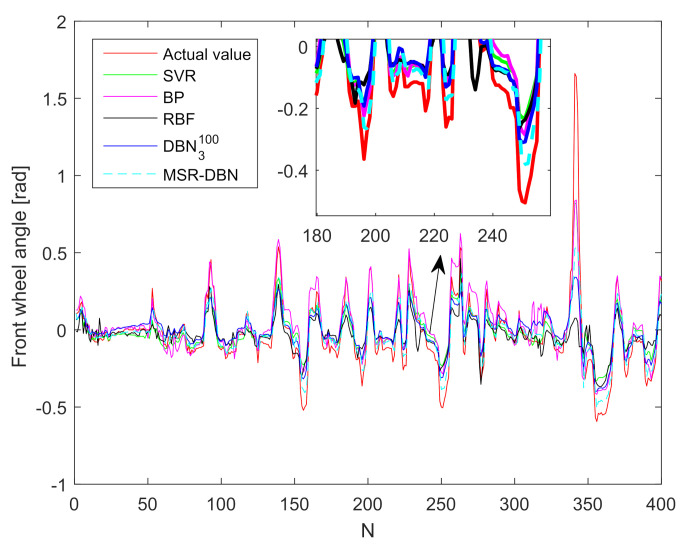
Prediction results of the front wheel angle based on different methods.

**Figure 8:**
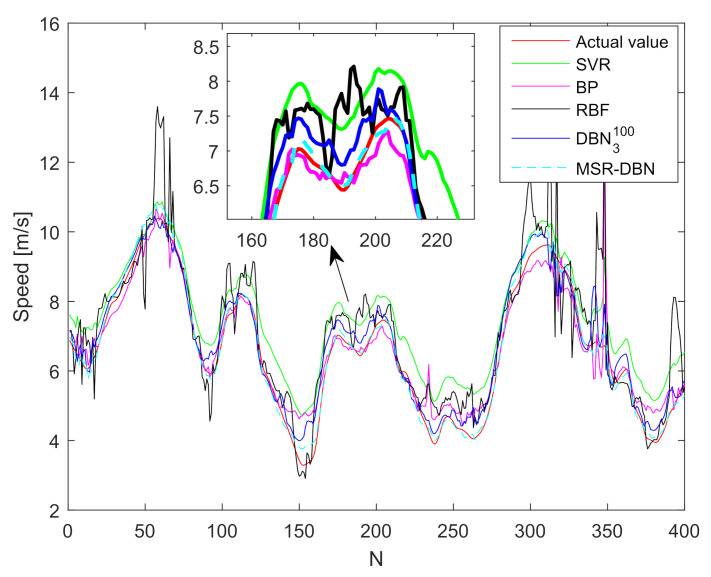
Prediction results of the speed based on different methods.

To demonstrate the performance of our improved MSR-DBN for driving behavior prediction, we analyze and compare the prediction results with ${\mathrm{DBN}}_{3}^{100}$, SVR, BP, RBF, and the actual values [24,44,54]. Figure 7 and Figure 8 show the prediction results of the front wheel angle and speed. We can see that these five prediction results have the same tendency with the actual value, indicating that all these five methods can predict driving behaviors for intelligent vehicles.

Figure 7 shows that SVR, BP, and RBF could achieve a relatively close performance. The prediction results based on ${\mathrm{DBN}}_{3}^{100}$ are closer to the actual values than SVR, BP, and RBF in the light of its deep architecture. However, the performance of MSR-DBN outperforms all of these four methods. For example, in the 250-th test sample, the MSR-DBN obtains the performance (−0.38 rad) that is the closest one to the actual value (−0.54 rad) than the others.

However, different results occur for speed, as shown in Figure 8. The RBF obtains the worst prediction performance, which even appears many big jumps for speed (such as the test samples 50 and 350). In addition, BP has more fluctuations while it obtains a satisfying performance for speed. However, the DBN achieves a better prediction performance, compared with RBF and SVR. Take the 190-th sample for example, SVR cannot obtain a prediction performance of speed as good as the front wheel angle, and the prediction result is approximately 7.35 m/s which is higher than the actual speed. The predictions of ${\mathrm{DBN}}_{3}^{100}$ and MSR-DBN (6.85 m/s and 6.5 m/s, respectively) are slightly larger than the actual one (6.45 m/s). Therefore, the MSR-DBN is more robust, compared with the other four models.

We also compare the prediction errors of MSR-DBN with the other four models to further illustrate the prediction performance, as shown in Table 7. Specifically, the RBF model obtains higher $\alpha_{\mathrm{RMSE}}$ and $\alpha_{\mathrm{MAE}}$ than SVR and BP. The ${\mathrm{DBN}}_{3}^{100}$ model obtains a larger error of front wheel angle, compared with other three models. However, the proposed MSR-DBN outperforms ${\mathrm{DBN}}_{3}^{100}$ with $\alpha_{\mathrm{RMSE}}$ (0.1165) and $\alpha_{\mathrm{MAE}}$ (0.0593), which is consistent with the results in Figure 7 and Figure 8.

For the prediction of speed, the SVR obtains the worst performance with $v_{\mathrm{RMSE}}=0.9142$, $v_{\mathrm{MAE}}=0.8447$, and $v_{\mathrm{MRE}}=0.1541$, followed by RBF and BP. Compared to SVR, RBF, and BP, ${\mathrm{DBN}}_{3}^{100}$ obtains a smaller error, and MSR-DBN outperforms ${\mathrm{DBN}}_{3}^{100}$. Thus, compared to ${\mathrm{DBN}}_{3}^{100}$, the MSR-DBN can not only improve the prediction performance of front wheel angle, but reduce the errors of speed, which shows a strong capability of predicting driving behavior.

### 4.4. Generalization Performance Analysis

**Figure 9:**
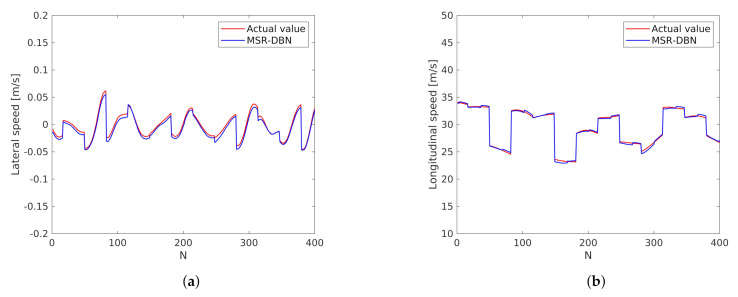
Prediction results on highD dataset. (**a**) Prediction result of lateral speed. (**b**) Prediction result of longitudinal speed.

In order to analysis the generalization performance of the MSR-DBN, test in different dataset is necessary. The highD dataset is a new dataset used by many researchers, it uses a drone to record the naturalistic vehicle trajectories on German [58]. It can provide higher-precision trajectory information by using state-of-the-art computer vision algorithms. Different from the urban road scenario data collected by ourselves, highD dataset is collected from the highway scenario. We can extract the position, speed and other information of the ego vehicle and surrounding vehicles from the dataset, but the steering wheel angle information is not provided. We choose the longitudinal velocity of x-axis as the speed, and the lateral velocity of y-axis to present the steering wheel angle. As shown in Figure 9, the MSR-DBN still has a good prediction performance.

## 5. Discussion

This paper presented a driving behavior prediction system with four sub-systems, data acquisition, data preprocessing, DBN prediction, and result analysis. This system can utilize multi-resource data including states of the ego vehicle, states of driver control, and states of surrounding vehicles. In addition, we used a systematic testing method obtaining an optimal DBN structure ${\mathrm{DBN}}_{3}^{100}$ and then developed a new DBN (called MSR-DBN, consisting of two sub-networks) by integrating a multi-target sigmoid regression to predict the longitudinal and lateral driving behavior simultaneously. A series of comparison results demonstrate that our proposed MSR-DBN can reduce the prediction errors of the speed and front wheel angle with more stable performance and higher accuracy for driving behavior.

Although our proposed MSR-DBN shows promising results, there still exist some work to improve further prediction performance, such as increasing more data. Additionally, more complex predictions will be conducted in the near future such as real-time behavior prediction, specific behavior prediction in different scenarios, and interaction behavior prediction.

## Figures and Tables

**Table 1:** Information of data collection.

Name	Parameter	Units
power unit	combustion engine with automatic transmission	-
travel distance	48	km
travel time	0.95	h
average velocity	50.27	km/h
collection time	3 to 3.57 (off-peak)	pm

**Table 2:** Prediction errors for surrounding vehicles based on DBN.

Type	Surrounding Vehicles∖Errors	$▵x_{\mathrm{RMSE}}$	$▵x_{\mathrm{MAE}}$	$▵y_{\mathrm{RMSE}}$	$▵y_{\mathrm{MAE}}$	$▵v_{\mathrm{RMSE}}$	$▵v_{\mathrm{MAE}}$
Case 00	Left front vehicle	0.1793	0.1223	2.6915	2.2376	1.3976	0.8935
	Front vehicle	0.2865	0.2139	2.3622	1.6879	1.2073	0.8054
	Right front vehicle	0.2875	0.2293	3.5747	1.9832	1.5416	1.1863
Case 01	Left front vehicle	0.4094	0.3794	3.9323	2.5273	0.9838	0.7563
	Front vehicle	0.1396	0.1036	3.6175	3.0658	0.5568	0.4278
	Right front vehicle	0.4806	0.4685	2.5734	2.0658	0.8483	0.6464
Case 02	Left front vehicle	0.2568	0.1527	3.6497	2.5632	1.5037	1.0070
	Front vehicle	0.2391	0.1758	9.7533	6.8699	1.3156	0.9809
	Right front vehicle	0.2899	0.2175	9.8397	5.5563	1.8377	1.2637
Case 03	Left front vehicle	0.1814	0.1068	6.2141	4.4135	1.8260	1.6235
	Front vehicle	0.1909	0.1352	5.1570	3.1565	2.0950	1.7989
	Right front vehicle	0.1901	0.1217	3.8158	2.2171	1.5692	1.3391
Case 04	Left front vehicle	0.2912	0.1947	11.3866	9.5590	2.0985	1.4397
	Front vehicle	0.2587	0.2040	9.0340	8.0025	1.5723	1.2682
	Right front vehicle	0.2613	0.1882	5.4806	3.4477	2.8110	2.4085

**Table 3:** Prediction errors for ego vehicle based on DBN with one RBM.

Type∖Errors	$\alpha_{\mathrm{RMSE}}$	$\alpha_{\mathrm{MAE}}$	$v_{\mathrm{RMSE}}$	$v_{\mathrm{MAE}}$	$v_{\mathrm{MRE}}$
Case 0	0.1935	0.1874	0.3055	0.2364	0.0172
Case 1	0.0946	0.0514	0.4668	0.3920	0.0654
Case 2	0.0323	0.0212	0.6665	0.5054	0.0317
Case 3	0.0617	0.0516	0.5971	0.4851	0.0395
Case 4	0.0279	0.0228	1.4551	1.2334	0.0968

**Table 4:** Prediction results of different learning rates for front wheel angle and speed.

Learning Rate	$\alpha_{\mathrm{RMSE}}$	$\alpha_{\mathrm{MAE}}$	$v_{\mathrm{RMSE}}$	$v_{\mathrm{MAE}}$	$v_{\mathrm{MRE}}$
0.1	1.0406	1.0118	15.1516	15.0381	2.5666
0.3	0.1029	0.0547	0.5516	0.4731	0.0814
0.5	0.0939	0.0493	0.5019	0.4335	0.0741
0.7	0.0946	0.0514	0.4668	0.3920	0.0654
0.9	0.0942	0.0494	0.4166	0.3381	0.0571

**Table 5:** Prediction results of different hidden layers for front wheel angle and speed.

Number of RBM	$\alpha_{\mathrm{RMSE}}$	$\alpha_{\mathrm{MAE}}$	$v_{\mathrm{RMSE}}$	$v_{\mathrm{MAE}}$	$v_{\mathrm{MRE}}$
1	0.0946	0.0514	0.4668	0.3920	0.0654
2	0.0900	0.0586	0.5763	0.4576	0.0878
3	0.1431	0.0811	0.3858	0.2965	0.0531
4	0.1171	0.0906	1.8569	1.5595	0.2867
5	0.1866	0.1310	1.5647	1.2909	0.2387
6	0.1427	0.1044	1.7684	1.4769	0.2716
Average	0.1290	0.0862	1.1032	0.9122	0.1672

**Table 6:** Prediction results of different hidden nodes in a layer.

Hidden Nodes	$\alpha_{\mathrm{RMSE}}$	$\alpha_{\mathrm{MAE}}$	$v_{\mathrm{RMSE}}$	$v_{\mathrm{MAE}}$	$v_{\mathrm{MRE}}$
32	0.1160	0.0766	1.8241	1.5283	0.2829
50	0.1279	0.0768	1.6475	1.3799	0.2543
64	0.1212	0.0717	1.0640	0.8687	0.1592
100	0.1431	0.0811	0.3858	0.2965	0.0531
128	0.2090	0.1555	1.2237	0.9832	0.1830
150	0.1426	0.0778	0.5932	0.4585	0.0778
200	0.1751	0.1260	1.5396	1.2659	0.2316
256	0.1614	0.1093	1.1938	0.9601	0.1720
Average	0.1495	0.0968	1.1840	0.9676	0.1767

**Table 7:** Error comparison for front wheel angle and speed based on different models.

Methods∖Errors	$\alpha_{\mathrm{RMSE}}$	$\alpha_{\mathrm{MAE}}$	$v_{\mathrm{RMSE}}$	$v_{\mathrm{MAE}}$	$v_{\mathrm{MRE}}$
SVR	0.1256	0.0813	0.9142	0.8447	0.1541
BP	0.1277	0.0860	0.5799	0.3684	0.0695
RBF	0.1806	0.1009	1.0403	0.6894	0.1090
${\mathrm{DBN}}_{3}^{100}$	0.1431	0.3858	0.0811	0.2965	0.0531
MSR-DBN	0.1165	0.0593	0.2067	0.1626	0.0259

## Data Availability

Not applicable.
